# Timelike curves can increase entanglement with LOCC

**DOI:** 10.1038/srep37958

**Published:** 2016-11-29

**Authors:** Subhayan Roy Moulick, Prasanta K. Panigrahi

**Affiliations:** 1 Indian Institute of Science Education and Research Kolkata, Mohanpur 741246, West Bengal, India

## Abstract

We study the nature of entanglement in presence of Deutschian closed timelike curves (D-CTCs) and open timelike curves (OTCs) and find that existence of such physical systems in nature would allow us to increase entanglement using local operations and classical communication (LOCC). This is otherwise in direct contradiction with the fundamental definition of entanglement. We study this problem from the perspective of Bell state discrimination, and show how D-CTCs and OTCs can unambiguously distinguish between four Bell states with LOCC, that is otherwise known to be impossible.

Entanglement and Closed Timelike Curves (CTC) are perhaps the most exclusive features in quantum mechanics and general theory of relativity (GTR) respectively. Interestingly, both theories, advocate nonlocality through them. While the existence of CTCs^1^ is still debated upon, there is no reason for them, to not exist according to GTR^2^,^3^. CTCs come as a solution to Einstein’s field equations, which is a classical theory itself. Seminal works due to Deutsch^4^, Lloyd *et al.*^5^, and Allen^6^ have successfully ported these solutions into the framework of quantum mechanics. The formulation due to Lloyd *et al.*, through post-selected teleportation (P-CTCs) have been also experimentally verified^7^.

The existence of CTCs has been disturbing to some physicists, due to the paradoxes, like the *grandfather paradox* or the *unproven theorem paradox*, that arise due to them. Deutsch resolved such paradoxes by presenting a method for finding self-consistent solutions of CTC interactions. The Deutschian model of CTCs (D-CTCs) impose a boundary condition, in which the density operator of the CTC system that interacts with a chronology respecting (CR) system is the same, both before and after it enters the wormhole. Formally,


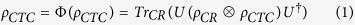


where *ρ*_*CR*_ is the density matrix for chronology-respecting system, *ρ*_*CTC*_ is the initial density matrix of the qubit traveling along the closed timelike curve, and *U* is the interaction unitary. Mathematically, this can be seen as nature finding a fixed point solution of the map, Φ, that depends on the chronology respecting system^4^.

Although a complete theory of quantum gravity is yet to be formulated, quantum information theorists have been studying the implications of the existence of CTCs and the nature of information with CTC-assisted models of computation. Recent studies of CTC-assisted models of computation, show them to be extremely powerful and be able to carry out non-trivial tasks, such as distinguish between non-orthogonal states^8^,^9^, clone unknown quantum states^10^,^11^, be able to signal superluminally^12^ and find a solution of any problem in the computational class PSPACE efficiently, in polynomial time (PSPACE = P)^13^.

These results could be due to the nonlinearity in the Deutschian model, that in turn could be due to the interactions between the past and the present. Another line of thought, as introduced by Pienaar *et al.* through Open Timelike Curves (OTCs)^14^ modeled the effects of such physical systems, where there was no interaction in the CTC, i.e. the Unitaries are Identity operators. Here, with entanglement between the qubits traveling along a timelike curve and an external chronology-respecting system, the self consistency conditions become,


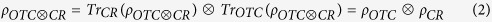


where *ρ*_*OTC*⊗*CR*_ is a bipartite system, and one of the systems is sent through the OTC. It can be seen that the OTC systems acts as a decorrelator. This trivially also violates the No-Broadcasting theorem. It was recently shown by Yuan *et al.*^15^, that such systems, with entanglement between the qubits traveling along a timelike curve and an external chronology-respecting system, could as well replicate the benefits of closed timelike curves without breaking causality.

Here, we turn our attention to understand the implications of existence of such physical theories of time travel on the nature of entanglement.

We begin by understanding the problem of Bell state discrimination and ask if it might be possible to distinguish between Bell states, using only local operations and classical communication (LOCC), given only a single copy of the state, from a set of four Bell States. We then try to understand its implications.

It is known, in the conventional model of quantum mechanics, it is possible to distinguish between any two Bell states using LOCC^16^, however it is impossible to deterministically discriminate between four or even three Bell states^17^. Here we take another look and study the problem of Bell state discrimination with the assumption of the existence of D-CTCs in nature.

*Bell state discrimination with LOCC* is defined as follows. Suppose a referee, Alice, prepares a single copy of a maximally entangled Bell state


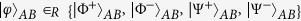


where 
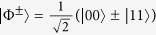
 and 
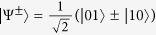
, and gives one qubit to Anita (|*φ*〉_*A*_) and one qubit to Babai (|*φ*〉_*B*_), who are spatially separated and allowed only local operations and classical communication. Their (Anita and Babai’s) objective is to determine which state was given to them.

One strategy Anita and Babai can pick would be the following.

Anita prepares a (known) state |*ψ*〉 = *α*|0〉 + *β*|1〉, such that 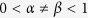, and perform a Bell measurement on her (known) state and her part of the local entangled qubit, |*φ*〉_*A*_, and classically communicates the measurement outcomes to Babai. Depending on the Bell state Anita and Babai were sharing, the decomposition can be given as follows, for each of the four possible Bell states.


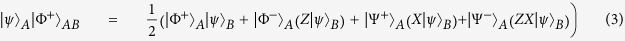



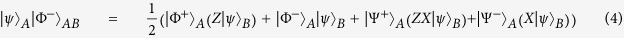



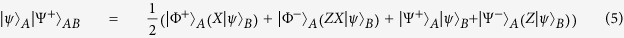



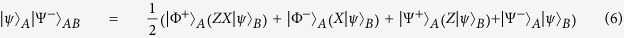


Based on Anita’s classical communication, Babai performs the necessary unitary operations on his share of the entangled qubit, as follows,


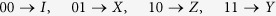


Here I, X, Y, Z are the Pauli operators, and [*cc* → *P*] represent Pauli *P* is applied by Babai on seeing bits *cc* from Anita. In a sense, they ‘force’ the teleportated states to pick up the unitary error associated with Anita’s Bell state measurement |Φ^+^〉_*A*_. Now all that remains for Babai is to mark out the unitary error his resultant state contains.

For this, we leverage two qubits traveling along a closed timelike curve (CTC systems) and chronology respecting qubits (CR system) containing the state and an ancilla, to distinguish between non-orthogonal states 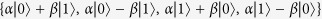, using a circuit^8^ as implemented in Fig. 1, where the unitaries are defined as


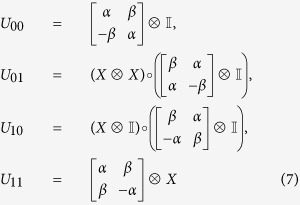


The circuit first swaps the CTC system with the CR system. Following that it performs a controlled unitary with the CR systems as the control and CTC systems as the target. Finally, it measures the CR system in the computational basis. The CTC system is a nonlinear system. This is because the outcome of *ρ*_*CTC*_, after the desired interactions, depends on the initial *ρ*_*CTC*_ (before the interactions) and the CR system *ρ*_*CR*_. Also, *ρ*_*CTC*_ (before the interactions) depends on CR system *ρ*_*CR*_. The objective here is to harness the non-linearity and exploit the two CTC qubits to effect the following map


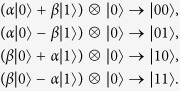


It can be seen, that the qubits traveling in a closed timelike curve remain unchanged, even after the implementation of the map, in each case, which is a necessary condition. Furthermore, these self-consistent solutions for the CTC qubits are unique, and satisfy Deutsch’s criteria as in eq. 1.

Let us understand one instance of what is happening in the circuit. Suppose the teleported state was 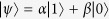. According to the desired interaction, it first swaps the information in the CR system and the CTC system. So, the CTC system now carries 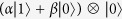. Since the CR system is now carrying |1〉 ⊗ |0〉, which the CTC system was initialized as, before the swap; unitary *U*_10_ now acts on the CTC system and results in the CTC system to become |1〉 ⊗ |0〉, before it disappears in the wormwhole. Thus Deutsch’s criteria for chronology respecting system is met and the qubits traveling along a CTC path remain the same both before and after the interaction.

What is essentially happening here is Anita prepares a known state, |*ψ*〉, and teleports it to Babai. The information of an entangled channel are not stored in the states but in the correlations. By teleporting the state, through the entangled channel, |*ψ*〉 is affected by the correlation. In a sense, the correlation of the entanglement gets downloaded in the state. By studying the change of the teleported state from the prepared state, it becomes possible to understand the nature of correlation in the channel. The circuit then, by measuring *b*_1_ and *b*_2_, of the chronology respecting qubits, learns which of the two conjugate eigenstates (through measurement *b*_1_) and the eigenvalue (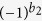), the teleported state is in.

The distinguishability of non-orthogonal states allows Babai to conclusively determine the Bell state that he shared with Anita. The corresponding Bell states, compared to the state identified by Babai, using the circuit as illustrated in Fig. 1, are shown in Table 1.

So, in conclusion, we could discriminate between Bell states with LOCC if there exists D-CTCs.

A more interesting question to now ask would be what happens if there were no unitary interactions in the CTCs. More concretely, can we distinguish the four Bell states if we had open timelike curves (OTCs) instead of CTCs? Here we show it is indeed possible, for an OTC assisted computer to distinguish between the four Bell states, while following a similar strategy with Anita teleporting a known state to Babai.

Suppose Anita prepares the state 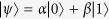, s.t., 0 ≤ *α* ≠ *β* ≤ 1 and 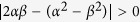. Then they follow the same strategy as above and Anita teleports the state to Babai. Babai now has the state 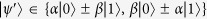 and needs to determine the exact state to conclude the Bell state, as previously shown in Table 1.

To do this, Babai uses the circuit depicted in Fig. 2. The unitary 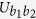 is chosen based on Anita’s Bell measurement outcomes *b*_1_*b*_2_. The unitaries are defined as


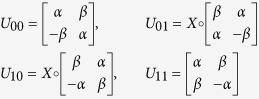


Babai prepares *N* ancillary qubits in the state |0〉. Following that he applies a C-Not gate, with the state |*ψ*〉 as control, targeting a fresh ancilla state each time. This correlates |*ψ*〉 with each of the N ancillaries. Each of the ancillaries are then passed through the OTC system. The OTC, as mentioned earlier, acts as a decorelator. Following that, Babai simply measures the states in the computational basis and correlates his measurement statistics and the Bell measurement outcomes he received from Anita earlier to identify the Bell state, as shown in Table 2.

To exemplify, suppose the unknown state shared between Anita and Babai was 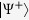. Anita creates the state 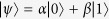, and performs a Bell measurement that admits the following decomposition,


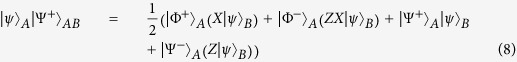


On her systems and (say) she sees outcomes 10. This she also communicates to Babai. Effectively, the state on Babai’s end is 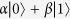 however this is unknown to them. According to the protocol, Babai now applies the unitary 
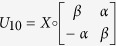
. This operation leaves the state as 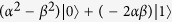. Following that, the CNOTs create a the state 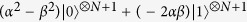. The OTCs acting as a universal decorelator, causes the resulting density matrix of each qubit passing through it as 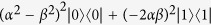. Upon measuring these qubits in the computational basis, (*α*^2^ − *β*^2^)^2^ of them outcome 0 and (−2*αβ*)^2^ of them to outcome in 1. Comparing this according to Table 2, we see, these measurement statistics could be produced by the state 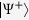. Hence determining the Bell state perfectly.

Hence we have shown by construction how to leverage the power of closed timelike curves and open timelike curves to distinguish between Bell states using only LOCC. But what does this say about entanglement in general? Consider the Smolin State, a certain four-party unlockable bound-entangled state^18^, shared between Anita, Babai, Charlie & Dan,


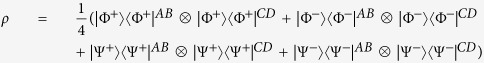


It can be seen that entanglement between AB and CD is 0, i.e.


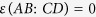


and the state is invariant under permutation. Thus,


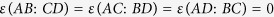


In other words, *ρ* is separable across the three bipartite cuts AB : CD, AC : BD and AD : BC^18^.

The logarithmic negativity^19^, *E*_*N*_, of the state *ρ*, in AC : BD cut, is


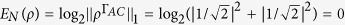


Since the distillable entanglement, *E*_*D*_, is upper bounded by logarithmic negativity^19^, we can say


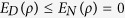


Thus distillable entanglement is exactly zero for a Smolin state.

However given the Smolin state to Anita, Babai, Charlie and Dan, which has zero distillable entanglement we can show that the using CTC-assisted computation, it is possible to create (increase) entanglement with just LOCC. Suppose Anita prepares a known qubit and teleports it to Babai. Following that Babai exploits his CTC-assisted computational circuit, as in Fig. 1, to unambiguously distinguish the Bell state without meeting. Following that, Anita and Babai classically communicate their Bell states to Charlie and Dan respectively, who now share a maximally entangled Bell state. Hence 1 − *ebit* was distilled between Charlie and Dan, using only local operations and classical communication, from the Smolin state through a D-CTC assisted computation. This shows, existence of D-CTCs would imply the possibility of creating entanglement using LOCC, which is otherwise impossible according to current formulation of quantum mechanics.

To conclude, our work here raises fundamental questions concerning the nature of entanglement in a world with timelike curves, that drastically changes our current understanding of quantum mechanics. An intuitive resolution to this might lead to support the chronology protection conjecture^20^, which loosely says such closed timelike curves cannot exist in nature^21^. If this were to be indeed true, such contradictions could indeed be evaded. However, we see that such conclusions arise even in chronology respecting open timelike curves. Due to this, our results also hold in a world with open timelike curves. So, in a sense, this may not be a problem with the Deutschian formalism, but a problem in nature and there might exist a non-linear extension of quantum theory^22^,^23^,^24^. A full theory of quantum gravity, we expect would perhaps resolve such challenges and contradiction between the implications of CTCs and laws of quantum mechanics and hope this work will help motivate further research.

## Additional Information

**How to cite this article**: Moulick, S. R. and Panigrahi, P. K. Timelike curves can increase entanglement with LOCC. *Sci. Rep.* **6**, 37958; doi: 10.1038/srep37958 (2016).

**Publisher's note:** Springer Nature remains neutral with regard to jurisdictional claims in published maps and institutional affiliations.

## Figures and Tables

**Figure 1:**
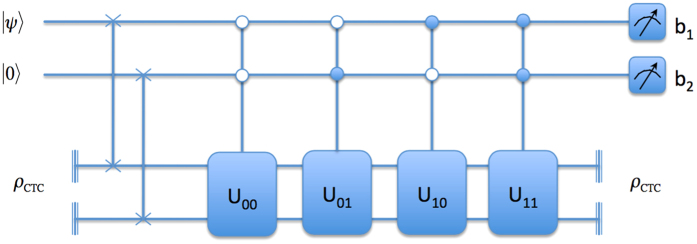
Circuit to distinguish between states {*α*|0〉 ± *β*|1〉, *α*|1〉 ± *β*|0〉} using Deutschian formulations of CTC.

**Figure 2:**
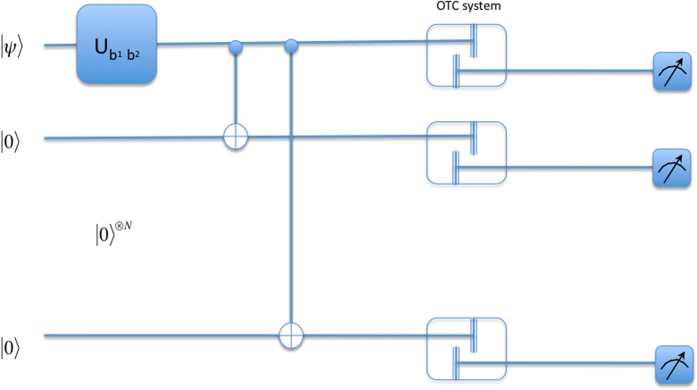
Circuit to distinguish between states {*α*|0〉 ± *β*|1〉, *α*|1〉 ± *β*|0〉} using OTC.

**Table 1:** Corresponding Bell States Anita & Babai share.

Measurements Outcomes, *b*_1_, *b*_2_	State Identified by Babai	Conclusive Bell State
0, 0	*α*|0〉 + *β*|1〉	|Φ^+^〉
0, 1	*α*|0〉 − *β*|1〉	|Φ^−^〉
1, 0	*α*|1〉 + *β*|0〉	|Ψ^+^〉
1, 1	*α*|1〉 − *β*|0〉	|Ψ^−^〉

**Table 2:** Lists the corresponding Bell States Anita & Babai share.

Anita’s Bell Measurements *b*_1_, *b*_2_	Babai Sees			
	All outcomes |0〉	All outcomes |1〉	*γN* outcomes result in |0〉 and *δN* outcomes result in |1〉	*δN* outcomes result in |0〉 and *γN* outcomes result in |1〉
0, 0	|Φ^+^〉	|Ψ^−^〉	|Φ^−^〉	|Ψ^+^〉
0, 1	|Φ^+^〉	|Ψ^−^〉	|Φ^−^〉	|Ψ^+^〉
1, 0	|Ψ^−^〉	|Φ^+^〉	|Ψ^+^〉	|Φ^−^〉
1, 1	|Ψ^−^〉	|Φ^+^〉	|Ψ^+^〉	|Φ^−^〉

The first column corresponds to Anita’s Bell measurements, and the first row lists the possible measurement outcomes for Babai who has an OTC assisted computer. Anita and Babai share the Bell state that is listed in the cell in row and column corresponding to their measurement outcomes. Here *γ* = (*α*^2^ − *β*^2^)^2^ and *δ* = (2*αβ*)^2^.
